# Kettle Logic

**DOI:** 10.1007/s10612-021-09569-x

**Published:** 2021-05-18

**Authors:** Mark Neocleous

**Affiliations:** Department of Social and Political Sciences, Brunel University London, London, UK

## Abstract

This article unearths the political logic of the police kettle. Rather than add to the mundane debate about civil liberties or models of policing, this article argues that the kettle reveals nothing less than the police war at the heart of modernity. This is a police war carried out as a logic of containment against the enemy within—within the kettle and within society. The kettle is a microcosm of the police war of containment.

## What is the Political Logic of the Kettle?

The news items are by now familiar enough: a crowd of protestors is held by police in a confined space, without food and water, with no access to toilets, no protection from the elements, and unable to leave until the police grant permission. The crowd has been kettled. The kettle might be held as a static mass, but the contained crowd can also be forced to move by the police—what one might refer to as a “mobile” or “wander” kettle. It can also take the form of a “bridge” kettle, such as that which occurred on the Pont de la Guillotière in Lyon, France, on October 20, 2010, and on Westminster Bridge, London, on December 9, 2010, where the bridge and the river helped the police form the kettle.

Discussions of kettling have centered on a combination of the impact of kettling on civil liberties, the lawfulness of the tactic, and the appropriateness of the tactic’s application given the incitement that the kettle produces (Douglas 2010; Ridler 2011; Rowan 2010; Taşkale 2012). The same questions emerge, time and again, reducing discussion to the tried and tested ways of thinking about policing—tactics and technologies, legalities and limits, and “models of policing.” Some commentators have treated kettling in terms of repressive police operations and the rise of what some call “command and control policing” (Vitale 2005). Kylie Bourne (2011: 191), for example, identifies a general “shift away from communicative models of interaction between individuals and governments to a more repressive and hostile relationship.” It goes almost without saying that, in one sense, the kettle is an example of the show-of-force that has been at the heart of police crowd control for some time. The kettle is, in the words of Roy Henry, former Commissioner of the Royal Hong Kong Police, a “projection of police units in an efficient, effective and formidable manner which creates an atmosphere in the riotous mobs of apprehension and awe which could be close to fear” (quoted in Northam 1988: 136). Such force, fear and awe are to be experienced not only by those in the kettle but also by those watching, for whom the kettle appears as a spectacle of absolute police power and hence a form of deterrence.

Yet is there really a categorical difference between “communicative models” and “repressive models” of policing? The “models of policing” approach is really rather trite and is certainly deeply uncritical. In this case, the distinction between “communicative models” and “repressive models” is really a version of the same dichotomy that arises time and again, such as that between the “paramilitary police model” (i.e., “repressive”) and the “community police model” (i.e., “communicative”). But such distinctions are part of the mythology of the liberal state (Neocleous 2014). Are not acts of repression, command, and control also acts of communication? “It wasn’t politics,” one of the French protestors of October–November 2005 explained, “we just wanted to tell the state something” (quoted in Bertho 2018: 26). Maybe the kettle communicates in the other direction: “it isn’t politics,” says the state, “we just want to tell the protestors something.” But what, then, is the message?

## “We Would Not Recognise the Term ‘Kettling’”

“A good metaphor is something even the police should keep an eye on,” Georg Christoph Lichtenberg once observed (1765–99/2000: 78). “Kettle” is one such metaphor. Symptomatic of how much the police keep an eye on this metaphor is the extent to which they repeatedly deny its very existence, for the “kettle” exists neither in police discourse nor in English law. Tellingly, what does exist is “containment.” Let us get to this point through some court cases.

In the case of a person been held in the Bishopsgate kettle in 2009, the divisional court (part of the High Court of Justice, but with two or more judges sitting) initially found the actions of the police to have been unnecessary and unjustified. The Court of Appeal, however, found the police actions to be lawful. In this case—*McClure & Anon v Commissioner of Police of the Metropolis* (2012)—the Court of Appeal held:

the decision to contain a substantial crowd of demonstrators, whose behaviour, though at times unruly and somewhat violent, did not of itself justify containment, was justifiable on the ground that containment was the least drastic way of preventing what the police officer responsible for the decision reasonably apprehended would otherwise be imminent and serious breaches of the peace.

In the case of *Castle & Ors v Commissioner of Police for the Metropolis* (2011), three student demonstrators between the ages of fourteen and sixteen were kettled in Whitehall, London, held for approximately seven hours in freezing temperatures, and surrounded by adults engaged in theft of property and the burning of street furniture. The court held that being contained by the police in these conditions was “justified by events occurring outside the cordon which required careful handling of those within the containment.” Similarly, in *Austin & Anon v Commissioner of Police of the Metropolis* (2009), a case in which Austin and three other people were held in a kettle on Oxford Street in London in 2001, the House of Lords found that such containment is lawful when police resort to it in good faith, for a legitimate purpose, is proportionate to the situation, and is enforced for no longer than is reasonably necessary. All these decisions were upheld in March 2012 by the European Court of Human Rights.

One thing to note is that what we have here is yet another series of examples of law doffing its cap to the police power: the police invent a tactic, and the law decides that the tactic is lawful. The point, however, is that the tactic in question was not “kettling,” but “containment.” Why “containment”?

Following the policing of the G20 protests in London, the Metropolitan Police were challenged by a Parliamentary Committee in May 2009 about the tactic of kettling, and we might benefit from considering what the senior police officers had to say. I apologize for the long quotations, all from the House of Commons Home Affairs Committee (2009), but because actually listening to the voice and language of the police power is often the best way of understanding policing, the passages are revealing, and I want to use them as a springboard into my wider argument.

One of the people questioned by the House of Commons Committee (the “Committee”) was Sue Sim, at that point, Lead Officer for the Association of Chief Police Officers (ACPO) in the United Kingdom (UK):

Mr. [Gary] Streeter: Could you just share with us the ACPO guidelines for the policing of public protests like this? I am interested particularly in kettling. …

Ms. Sim: Firstly, I do not understand the term ‘kettling’. Kettling is not a British policing public order tactic, it is something that has been created apparently in the media.

Mr. Streeter: What do you call it when you group people together in this way?
Ms. Sim: I would call that containment.

Note the disingenuous nature of Sim’s comments. She says she does not understand the term, but clearly knows enough about it to identify it as a media creation that refers to a specific police tactic. Unsurprisingly, this line of questioning went nowhere. But later in the discussion, another committee member, Labour MP Karen Buck, comes back to the point:

Ms. Buck: Going back to the issue of kettling as a term, as a concept, it is something that has entered discourse in terms of crowd control probably since the May Day demonstrations at the beginning of the decade. In an earlier answer you kind of rejected it as a term. Are you saying, really, that this is a media invention and that actually there has been no change in the tactics of crowd control?
Ms. Sim: Kettling is not a term that is contained within any policing manuals or with any policing concept. The issue of containment … has been a tactic for a long time. On the issue of kettling, I do not actually understand how that has been come into the terminology because it is not something that we would accept; “containment is.”

Another Committee member, Bob Russell, a Liberal Democrat MP, then takes up the issue:

Bob Russell: I wonder if you could give us the date when this system came in? … Like you, I have no idea where the term ‘kettling’ comes from, but the word ‘kettle’ does strike me as being very close to something that can boil over, and I suspect that this is what has happened.
Ms. Sim: Yes, but it is not within police terminology.
Bob Russell: The point I am trying to get at is: is this a completely new method of policing? It strikes me as being something different from what it has been historically and I want to know at what point it changed to what we have got today?
Chairman [Keith Vaz]: Mr Russell needs to know the date when kettling began.
Ms. Sim: We do not kettle, Mr Vaz.
Chairman: The date it began even though you do not do it. This is a media term, is it, the word kettling? It is not a police term.
Ms. Sim: I believe it to be a media term.
Chairman: You do not arrive on the scene and say, ‘Let us kettle these people’?
Ms. Sim: No, we do not.

The same line of discussion continues in the Committee’s questioning of Sir Hugh Orde, Chief Constable, and Duncan McCausland, Assistant Chief Constable of the Police Service of Northern Ireland.

Chairman: Can you specifically tell us about what we have heard, which Mr Russell and others have raised, about kettling and kettles? Where does this term come from and what does it mean? Is it a recognisable police term or is this a Sky News term?

Sir Hugh Orde: I have absolutely no idea where the term came from. Issues of containment are very clearly tactics. … Duncan, do you want to touch on the tactics that we would use around containment?

Mr. McCausland: We would not recognise the term ‘kettling’. … I believe it has been something created by the media about a kettle being on the boil. We would use clear containment tactics, that you heard and that Deputy Chief Constable Sims mentioned, but our role in terms of containment, Chairman, has been to potentially diffuse the situation and allow protesters and people to move away from the area that they are potentially wanting to get into. Sir Paul Stephenson [Commissioner of the Metropolitan Police Service] observed that ‘we think that [kettling] is an entirely inappropriate term’.

In an ensuing session a week later (May 19, 2009), the Committee questioned Sir Paul Stephenson and Commander Bob Broadhurst, the “Gold Commander” of the Policing and Security operation of the G20. Committee member Bob Russell returns to the matter:

Bob Russell: Commissioner and Commander, this is the second session we have had where the term ‘kettling’ or ‘kettle’ has been used. I find it offensive. I do not know where the term has come from. The police have stated it is not terminology they use. I wonder if, first of all, you could tell us what your terminology is.
Chairman: Are you implying that ‘kettle’ is not a British term?
Bob Russell: It is something, Chairman, that in my many, many years in public life, and as a former court reporter, I have never heard of until relatively recently. So I am just wondering where the term came from.

Sir Paul Stephenson: It is not a term we use; it is not a term we favour; we – and I think it is in the ACPO manual—use the term ‘containment’, and that is what we will continue to use because that accurately describes what the tactic is.

The law also follows this police insistence on referring to “containment,” not “kettling,” as can be seen not only in the numerous legal judgments on the issue cited above, but by the fact that the senior police officers responsible for deciding the duration of the kettle and how people will be dispersed are called “containment managers.”

The police insistence on “containment” as the language and logic at stake has a long history: “since the manuals began,” as Sim put it. The “show-of-force,” cited above, as the heart of the kettle, appeared in the discussion of containment in a police training manual by Colonel Rex Applegate, published in 1969, which felt no need to explain what “containment” is other than describing it as a police tactic (Applegate 1969). To use a more recent example, *Keeping the Peace: ACPO Manual of Guidance: Public Order Standards, Tactics, and Training Manual (*the *“Manual”)*, contains no reference to “kettles” or “kettling,” but plenty of references to a tripartite logic of “containment, dispersal, arrest,” and various styles of cordon as different containment techniques. The *Manual* is clear that, along with technologies (e.g., the armored vehicle) or tactics (such as the roadblock), a cordon is always intended as “show-of-strength” (ACPO Public Order Working Group 2004: 52, 124, 199–204). The British police thus define the tactic of “containment” as “strategic incapacitation” (HMCIC 2009: 43).

So important is this idea of containment to the state that we should observe two important features. First, in the case of *Austin*, Lord Neuberger made the argument that protestors can be regarded as having given their *consent* to being contained:

If imputed consent is an appropriate basis for justifying confinement for article 5 purposes, then it seems to me that the confinement in the present case could be justified on the basis that anyone on the streets, particularly on a demonstration with a well-known risk of serious violence, must be taken to be consenting to the possibility of being confined by the police.

In other words, “consent to being confined could be *imputed to the people concerned*” (emphasis added). Now, Lord Neuberger’s position was not quite the position of the Court. But the way he frames his view is in line with the Court’s position and this is telling because of his use of the legal notion of “imputed consent.” “Imputed consent”—that is, consent between two parties who are treated by law *as if* they had consented—in a public law setting concerning a “crowd” (and hence, by implication, involving a very different notion of voluntariness) is a means of insisting that *some* form of consent to be contained can be identified among those contained. The disobedience of our protest is interpreted as a sign of our consent to *de jure* containment and *de facto* imprisonment. (I use the term “imprisonment” deliberately for reasons that will become clear.) Second, the state holds the view that the containment of people can take place simply on the grounds of “a mere speculative danger,” as Her Majesty’s Chief Inspector of Constabulary’s (HMCIC 2009:61, 89) document, *Adapting to Protest*, puts it. In other words, containment can be used in response to *potential* as well as *actual* disorder.

We are thus beginning to be able to identify some of the features of kettle logic: the kettle is an act of *pre-emptive containment to which those being contained have already consented*. What is also clear, however, is that the logic of the kettle requires an understanding of the logic of containment.

“Containment” as an act of holding has a long history, going back to the seventeenth century. The *Oxford English Dictionary* (*OED*) offers, as the first appearance of the word, a translation published in 1619 of an Ancient Greek book on wealth, with the sentence “let us now see, if there be not as good meanes of vertuous containment, as well in the dayes of peace as of warre.” The idea then gains some momentum in the seventeenth century. In political texts, such as Hobbes’s *Leviathan*, the move from the state of nature to the political state is a mechanism to *contain* every person. Hobbes is the philosopher of motion par excellence (“life is but a motion,” as he states at the very beginning of *Leviathan*), yet he has us leave the state of nature into a state in which we are no longer simply bodies in motion but are now bodies *contained* and hence *constrained* by sovereign power. The logic of security lies in the sovereign’s power to contain us despite life being movement (1651/1991: 123).

The idea of “containment,” however, really comes into its own only much later, during the Cold War. The *OED* cites an article from *The Partisan Review* in 1947 that uses the word, “containment,” but the first key usage is in “The Sources of Soviet Conduct,” an article which appeared in the journal *Foreign Affairs*, that same year, under the authorship of “X”—a pseudonym of George Kennan—one of the leading architects of the United States’ national security state (X [Kennan] 1947). It was Kennan who gave the first explicit statement of the meaning of “containment,” and he did so by placing it at the heart of the emerging security doctrine. Both John Lewis Gaddis (1978) and Paul Chilton (1996) have shown that from 1947 onwards, the fundamental outlines of a strategy of containment received the endorsement of the highest authorities, was explicitly stated in many documents, presupposed in many others, and became a regular feature of discussion in intellectual circles. In this context, containment was conceived as a *political* and not just *military* act. The issue for the newly emerging national security state “was not the containment by military means of a military threat, but the political containment of a political threat,” as Kennan (1967: 358) later put it. Reflecting later, still, on his use of “containment” in the “X” article, Kennan commented (1986: 25–6) that.

in no way did the Soviet Union appear to me, at that moment, as a military threat to this country. Russia was at that time utterly exhausted by the exertions and sacrifices of the recent war. Something like 25 million of its people had been killed. The physical destruction had been appalling. … To have remobilized the Soviet armed forces at that time for another war effort, and particularly an aggressive one, would have been unthinkable.

He continued:

In these circumstances, I reiterate, there was no way that Russia could appear to me as a military threat. … So when I used the word containment with respect to that country in 1946, what I had in mind was not at all the averting of the sort of military threat people are talking about today. … What I *did* think I saw—and what explained the use of that term—was what I might call an ideological-political threat. … There seemed to be a danger that communist parties subservient to Moscow might seize power in some of the major Western Europe countries, notably Italy and France, and possibly in Japan. And what I was trying to say, in the article I am talking about, was simply this: ‘Don't make any more unnecessary concessions to these people. Make it clear to them that they are not going to be allowed to establish any dominant influence in Western Europe and in Japan if there is anything we can do to prevent it’. … This, to my mind, was what was meant by the thought of ‘containing communism’ in 1946.

As such, insofar as containment involved the “keeping in place” of the Communist threat in the form of the Soviet Union and a “keeping in place” of the Communist movement beyond the Soviet Union, so it also involved a “keeping in place” of the enemy at home. As Andrew Ross (1987: 331) points out, there are two different meanings of containment:

One which speaks to a threat *outside* of the social body, a threat which therefore has to be isolated, in quarantine, and kept at bay from the domestic body; and a second meaning of containment, which speaks to the domestic *contents* of the social body, a threat internal to the host which must then be neutralized by being contained or ‘domesticated’. [emphasis in original]

Security of the social order was to be achieved by containment of opposition *at home* as much as it by keeping the Soviet threat *at bay*. Containment may well appear to have started as a foreign policy, but its real power lay in being a rhetorical device depicting a world in which law-and-order is *always* already under threat from an enemy *within*.

Containment, then, quickly and easily came to operate as a key police category: the containment of international communism was to be administered by the “four policemen” overseeing the international order of states, while the security of the capitalist order at home was to be administered through the containment of anything that was thought to threaten the social order—organized labor, the demands for recognition and equality by the women’s liberation movement, Black liberation and the political struggles of racial minorities, and the student movement of the 1960s. By the 1970s, “containment” had become fundamental to police power in the widest possible sense of that term. American cities, such as Los Angeles, would implement major redevelopment plans for the impoverished area known as “Skid Row,” covering fifty square blocks and several thousand homeless individuals, vulnerable persons, and “problem” people, and would openly call it a “Containment Plan.” Likewise, in the United Kingdom (UK), containment was being widely used to police the working class, to which we shall turn shortly. Playing heavily on our everyday semantics surrounding security and order, containment came to function as a microphysics of power resonating through the personal, as well as the political, lives of citizens, right down to the health of the social body (in the form of the “containment” of viruses—from Communism to COVID-19, we might say) (Neocleous 2022). Containment thus functions as a police operation *par excellence* (Neocleous 2008).

“The containment of social change is perhaps the most singular achievement of advanced industrial society,” observed Herbert Marcuse (1964: xii) at the height of the Cold War. Marcuse was grappling for a way of understanding how it is that capitalism and its technological rationality appears capable of holding back the possibility of social change. Combining the powers of welfare and warfare, developing mechanisms of total administration, and finessing a system of countervailing powers that could cancel each other out, capitalism appears able to combat and defeat any historical alternatives that might emerge—from either within or without. Marcuse grasped that the foreign policy of containment is, in fact, an extension of domestic containment. A logic of containment was now the dominant logic of capitalist states—the pacification of the people through the reproduction of the social order and the forestalling of any threat to that order. Perhaps, then, the kettle as the microphysics containment is a specific expression of the far wider pacificatory logic underpinning bourgeois order? With this in mind, we can now deepen our characterization of kettle logic.

## “Thou Shalt not have the Gods of Other Nations”

A remarkable feature of contemporary protests is the fact that they are configured as *battles*: the “Battle in Seattle” (1999), the “Battle of Genoa” (2001), the “Battle of London” (2010). Such militaristic language is more appropriate than the protestors realize, not only because sovereign control of territory and space is key to the logic of war, but also because of the history of kettling in the military strategy of encirclement: *Kesselschlact* is the German word for “military encirclement.” This is a process in which troops surround and isolate an enemy force. Perhaps the most famous example is the battle for Stalingrad between August 1942 and February 1943. Indeed, in his book, *Stalingrad* (1999), Anthony Beevor observes that the question of how the *kessel* might be *policed as an act of war* and *fought within as an act of police* was central to Nazi strategy. Scott Sørli (2014: 149), in turn, claims that “the line between kettling and war is a fine one.” There is, in fact, no such line. Just as the supposed line between war and police turns out to be a myth (Neocleous 2014), so the kettle needs to be understood as a police tactic of the war power and a war tactic of the police power.

The war to enclose space and to call this enclosure lawful is one of the hallmarks of capital as it seeks the capture and possession of property, bodies, and life itself. Once space has been enclosed in this way, fundamental tensions are generated between stability and movement, partition and clearance, segregation and manoeuvre. Capital needs (the) police power to manage such tensions. This is one reason why, for all capital’s bravado about movement—about its own ability to nestle and settle everywhere and about the “freedom of movement” it brings to people—the world is nonetheless also the site of so many barriers, bars, borders, cordons, fences, fortifications, gates, walls, wires, and other such paraphernalia of territory. Such tensions manifest themselves in everyday police operations, such as keeping people in place and making them move—a version of the police ability to use opposite commands: both “moving too quickly” and “moving too slowly” can count as suspicious behavior and the grounds for a stop-and-search. And yet, remaining stationary is equally no solution for those subject to police power because this can count as “lingering” or “loitering” and hence is equally suspicious and grounds for a stop-and-search. Unless, that is, *one is forced to remain stationary by the police*.

Forcing people to remain in the same space turns the kettle into a police cell (or holding cell) on the street—a mobile prison the walls of which are made from the actual physical bodies of the police, their truncheons, shields, horses, dogs, and armored vehicles. Recently added to this list is the mobile steel police cordon, around three metres high, which can be folded to create metal holding cells enabling the police to kettle people into even smaller spaces. There is no exit from this prison other than with the permission of the police. When the police grant you permission to leave, it becomes an offense *to refuse*.

In giving opposite commands and sending people this way and that—“Move!” “Don’t move! “Move faster!” “Slow down! “Go home!” “No, you can’t go home!”—the kettle not only demoralizes, but also incites. This incitement is important to what has been called kettling’s affective dimension (Philippopoulos-Mihalopoulos 2015; Wall 2019). But what does it incite? Rage. The kettle produces the very rage that it wants to police, thereby justifying the very use of the kettle. We need to pause here on this point.

*Kettel* comes originally from the Old English *cetel* and *cietel*, and in Middle English, we also find *ketel* and *chetel*, influenced by an Old Norse word *ketill*. The Old English *cetel* is close to the West Frisian *tsjettel* (“kettle”) and the Dutch *ketel*, but is based on the Latin *catillus*—“small bowl”—diminutive of *catinus*, connoting “deep bowl,” or larger vessel for cooking or serving food, and hence giving us “kettle,” as well as “cauldron.” Other terms also point in this direction: the Swedish *kittel* is closer to “cauldron,” as is the Russian *кoтёл* and *kotjól* (“boiler” and “cauldron”). *Kesselschlact*, mentioned above, is the German word for military encirclement, which means literally “cauldron battle.” The kettle as cauldron and boiler reminds us that kettles are containers in which things *get heated*. In the kettle, things are brought to a boiling point through intense heat and a high degree of agitation. The kettle, therefore, *contains* materials in order to *bring them to a boil*. Yet also, conversely, and in the words of the Chief Constable cited above, the very word “kettle” indicates that it is something that might boil *over*. Hence, the kettle is *meant* to bring things to the boil but, also, to *prevent the contents from boiling over* in a dangerous way. The kettle is a cauldron for boiling rage.

The word “rage” enters the English language around 1300, connoting madness or insanity, a fit of frenzy, an anger or wrath, and a fierceness in battle. It appears to stem from the Old French *rage* or *raige*, meaning “spirit, rage, passion, madness, fury” but also from the Medieval Latin term *rabia*, from Latin *rabies* (“rage, madness, fury”), related to *rabere* “be mad, rave,” which is also the source of the Old English *rabbian*—“to rage.” In other words, “rage” suggests that there is a creature who needs to be contained, not just because it is angry or frenzied, but also because it is contagious, mad, or rabid.

For this reason, among others, the kettle involves treating people like animals. The language of “herding” into “pens” is common in police discourse, and the kettle here comes into its own. Containment is a tactic “to herd the crowd into a pen, known as ‘the kettle’,” observed one former senior Metropolitan Police officer in 2010 (quoted in Joyce 2010). Herding into pens is what one does with animals. So too is “keeping.” Animals are “kept” in pens. Animals are “kept” in spaces. Animals are simply “kept.” Humans can be equally “kept” in this way, equally contained and, from the police perspective, necessarily so. The keeping of the people in the kettle is a reminder of their status as animals in the eyes of the police, whether as livestock or beast. “Man is always regarded by the police … as a species of wild animal and treated as such,” a character comments in Friedrich Schiller’s play *Die Polizey* (1799–1803/1982: 93)—an idea that runs through bourgeois thought in general, as Marx (1844/1975: 242) points out in his *Manuscripts*: “political economy knows the worker only as an animal.” The issue, of course, is not simply that the police, like the bourgeois class whose order it fabricates and whose law it enforces, regard us as animals. Rather, the issue is that keeping people in a kettle is a reminder that *those who have penned them in are their masters*. As the trope of animality makes clear, the containment produced by the kettle is part of the taming of the creature being held within, a training in how to accept their capture inside the cordon and, ultimately, their capture inside capital. To accept, that is, their containment in general.

Keeping other living creatures captive is a way of holding over them a death sentence. The sentence might be suspended over and over, but we are never allowed to forget that it exists. The police kettle thus contains the ultimate power of the master—the threat of death for any living creature within it. This takes us back to the kettle as a form of police war, only this time in the form of new techniques of warfare, such as the kill box.

In a memorandum to US Secretary of Defense Donald H. Rumsfeld in February 2005, James A. Thomson, president of the RAND Corporation, suggested changes to traditional forms of coordination between air and ground support, in what he described as the “long war.” The changes had at their core a system of “kill boxes”—a technique that had been first used in January 1991 during Operation Desert Storm. Thomson’s memorandum suggested that “kill boxes can be sized for open terrain or urban warfare, and opened or closed quickly in response to a dynamic military situation” (quoted in Belote 2006: 63). Within a few months, the US Army, Navy, Air Force and Marines produced a new field manual called *Kill Box: Multi-Service Tactics, Techniques, and Procedures for Kill Box Employment* (2005). This manual cited the Department of Defense definition of the “kill box” as “a three-dimensional area reference that enables timely, effective coordination and control and facilitates rapid attacks,” but went on to say that despite this definition, there was no formal kill box doctrine or procedures, tactics, and techniques; this was the purpose of the new manual. From then on, the war power, in general, set about “bringing the box into doctrine,” as one 2004 Army monograph has put it (MacGregor 2004). “Kill box management” has since become central to the rules of engagement of twenty-first century warfare.

Everything said about the kill box might equally be said about the kettle. Any force using the box must have “profound technological and logistical advantages over its enemy,” including “a sophisticated web of logistical, bureaucratic, and technological expertise to implement,” as Scott Beauchamp (2016) has described. But as Beauchamp (2016) spells out, kill-box strategy is now used in conflicts *within* states rather than between them:

In recent years, kill-box strategy has shifted: They are now used in conflicts that are not between two states, but rather within states against terrorists and fighters who aren’t members of any particular country’s military. With this change, two things have started happening. First, kill boxes have materialized in places the local population might not expect. And second, kill boxes have been used in conjunction with disposition matrices … to target people whose ‘pattern of life’ fit the parameters of an algorithm, rather than specific individuals.

Beauchamp claims that “kill boxes are only used in places that are very different from the United States; military forces would never initiate a kill box in Manchester or Ann Arbor.” Yet, given the nature of a war against “patterns of life” considered insurgent, subversive or threatening of order, and given that the central logic of the kill box is to *govern space*, Beauchamp’s claim is more than a little dubious. The kill box is nothing less than a space defined as a target area over which violence can be exercised, replicating the very nature and purpose of enclosing space in the first place. At the heart of the kill box technique is the combination of the control of the space of battle—especially from above, which is why the technique has been at the heart of the development of the drone (Chamayou 2015; Weber 2017)—and an ongoing risk assessment about the degree of violence to be exercised over that space. The purpose of the kill box is to *trap* the enemy, *herd* it, *keep* it and *exert violence over it*.

The language of “herding” and “trapping” animals in the UK is commonly found in relation to the policing of football supporters in the UK. At one point, this involved literally *caging* supporters in pens. During a match between Liverpool and Nottingham Forest at Hillsborough Stadium on April 15, 1989, an overcrowding of football supporters in the section of the ground allocated to Liverpool supporters was facilitated by a police decision to order a further gate to be opened to allow in yet more supporters. The supporters were then unable to escape the overcrowding that ensued, due to the cages in which they were being contained. The resulting 96 fatalities and 766 injuries means that it is by far the worst “disaster” in British sporting history. The “Hillsborough Disaster” was due largely to the herding of football supporters in cages, and subsequent inquiries, most significantly the report by the Hillsborough Independent Panel (HIP) led by Phil Scraton, have made clear that the deaths occurred because the operative police principle was the *containment* rather than the *welfare* of fans (HIP 2012; Scraton 2016). The submission to the inquiry by the Ambulance Service present in the stadium held that the evidence that supporters were being crushed to death “was evident to anyone whose mind was not conditioned by the need to contain supporters within the central pens” (HIP 2012: para. 2.4.72). Only when it became clear that supporters were being *killed* did the police strategy move from containment to rescue, by which point it was too late. The kettle once again contains the dead enemy.

Enemy? At a football match? In the years leading up to the events of April 15, 1989, the British state had been well aware that the construction of football grounds as a series of “pens” or “cages” from which supporters would not be able to escape from police containment was liable to lead to a disaster. The *Wheatley Report on Crowd Safety at Soccer Grounds* (1973) had recommended a required time for exiting a stadium as eight minutes or fewer and the restructuring of entrances and exits to this end. This was updated in 1986 in the *Guide to Safety at Sports Grounds* (the “*Guide*”)—itself a response to the fire at Bradford Stadium in 1985 (in which 56 people died having been unable to escape their “containment” in the stand that was on fire). The *Guide* recommended a metering system at turnstiles, intercommunication systems across grounds, and contingency plans for evacuation. Yet, as Ian Taylor (1989) and Phil Scraton (2016) have shown, all such recommendations with respect to architectural design and the welfare of supporters were overridden by the state’s obsession with the containment of the mass of supporters and the wider penal discipline of individual offenders. The extent of this obsession is illustrated most explicitly by *The Popplewell Report* (the “*Report*”), the official inquiry into the fire at Bradford. The *Report* included expert evidence on the dangers posed by accumulated litter, the velocity of fire, and technical aspects ground construction. From this, the *Report* made a series of recommendations completely unconnected to fire and its hazards, and instead proposed revising the criminal law in respect of the police powers to search spectators entering grounds. The logic of the *Report* only makes sense, Taylor suggests, as an example of the ways in which issues of crowd safety and provision were displaced by a much wider logic of containment and penal discipline. The more general point is that this was, without question, a product of the wider political moment in which the Thatcher regime had defined organized working-class political movements, such as trade unions, in no uncertain terms—“The Enemy Within.” What took place at Hillsborough, then, was not a “sporting disaster,” but the result of the *physical containment* of an overwhelmingly working-class football audience as an instantiation of the British ruling class’s desire for the *political containment* of the working class. It is, therefore, unsurprising to find that three decades later, the mass killing in the kettle at Hillsborough continues to generate a powerful rage.

The kettle produces boiling rage by first and foremost producing the very crowd that is to be enraged. Foucault (1977: 201) comments that in bourgeois order, “the crowd, a compact mass, a locus of multiple exchanges, individualities merging together, a collective effect, is abolished and replaced by a collection of separated individualities.” Others have made the same point directly about the kettle: “when the police talk about splitting up crowds and dividing different elements, they demonstrate their … sense of the crowd as merely a collection of individuals” (Wall 2014). This makes sense if, as David Correia and Tyler Wall tell us (2018: 211, 213), “cops are scared of crowds,” for the crowd is, in the police view, “always about to explode, always a riot about to happen.” Yet, if this is true, *why create a kettle?* Why create the very thing that appears to abolish individualities and generate something that is always about to explode? The threat contained in the kettle is a threat produced by the kettling. The kettle *fabricates the very crowd* that the police need to contain; the police power *creates the collective threat to be contained*. The question asked by Al Sandine (2009: 117) in his history of the American crowd is always the question to ask: *who owns the crowd?* And in the kettled crowd, the police answer is clear: *we own you*. The kettle is a form of crowd creation as an act of sovereignty, in order that a yet greater act of sovereignty might then legitimately follow: the possibility of death or, if not death, then demoralization, discretionary punishment, identification for police records, and the humiliation of being sent home tired, hungry, and smelling of piss.

Yet, conversely, and despite fabricating the very crowd to be kettled, the kettle, in fact, denies the crowd the attributes of being a genuine crowd. In *Crowds and Power* (1962: 29), Elias Canetti observes that the crowd always wants to grow, loves density, and needs a direction. The kettle, in contrast, is a crowd created by an act of police power and thus its density, direction and tendency are always already controlled by the police. It is a crowd that is also somehow not a crowd—a crowd created to be dispersed, a crowd fabricated to be destroyed, a crowd structured in order to be dominated by the state and ultimately defeated in its very constitution as a crowd. *The kettle is a crowd constituted in order to be contained*. The message is clear, already cited above in the words of the former Commissioner of the Royal Hong Kong Police: feel the apprehension, awe, and fear. This is what incarceration feels like. This is what immobilization feels like. *This is what can be done to you*. In that sense, the kettle is once more an expression of the state’s absolute power: I bring you into existence; I own you; I can destroy you.

What the kettle also brings into existence is the lawbreaker. Because the kettle operates as a form of detention and preparation for arrest, because it creates a crowd in order to incapacitate it, because it incites the crowd to re-capacitate itself as a crowd, and because it provokes individuals into carrying out arrestable infractions of the law, one of the kettle’s most creative acts is to provoke lawbreaking. The protestor-criminal produced by the kettle becomes the problem criminal-protestor for which the kettle is said to be needed. The lawbreaking is then used by the police power to justify the kettling as a pre-emptive act. The kettle becomes its own justification. The intention behind the kettle thus seems to be a provocation to individuals and the crowd as a whole to disrupt the very thing that has put them there in the first place—to prompt their further arrest or, should such provocation fail, to remind them of their complete subordination to the police power. Once again, the rage of the kettled becomes predictable.

In the history of bourgeois thought and as the above etymology has made clear, rage coincides with madness. For Hobbes, for example, rage has its roots in excessive passions: pride combined with anger is rage; excessive and habitual desire for revenge becomes rage; excessive love combined with jealousy becomes rage; excessive opinion of oneself combined with envy becomes rage; having one’s vehement opinions contradicted by others becomes rage. But the excess is always in danger of becoming political. “When many … conspire together, the Rage of the whole multitude is visible enough,” says Hobbes (1651/1991: 54–55); when rage engulfs the mass, the madness of the enraged individual becomes “Madnesse in the multitude.” Hence, the real issue with rage is that it risks grasping the multitude and encouraging them to *challenge the very power that has offered them security*. As Hobbes (1651/1991: 54–55) puts it:

For what argument of Madnesse can there be greater, than to clamour, strike, and throw stones at our best friends? Yet this is somewhat lesse than such a multitude will do. For they will clamour, fight against, and destroy those, by whom all their lifetime before, they have been protected, and secured from injury.

In a sense, the rage of the multitude is a sign that the multitude has lost its mind, most obviously when it targets the very thing that provides it with security and smashes through the purported rationality of the system. The police power steps in to crush the rage, reassert the system’s rationality, and thereby justify its existence as police. The kettle is, in this sense, indicative of nothing less than *the necessity for the police of the whole system* and a *political project for containing any challenge to that system*.

In Chapter 30 of *Leviathan*, Hobbes (1651/1991: 233–234) describes the desire to challenge the power that offers us security as a political sin against the sovereign equal to a breach of God’s first commandment: “This desire of change, is like the breach of the first of Gods Commandements: For there God says, *Non Habebis Deos Alienos*; Thou shalt not have the Gods of other Nations.” Truth be told, Hobbes is not really worried about people being tempted by the Gods of other nations. Rather, he wants to discourage people from thinking that “the prosperity of a People” comes from aristocracy, democracy or any other such forms of rule. Prosperity comes from “the Obedience and Concord of the Subjects.” The most dangerous other “nation,” then, is the one which people think they can create through disobedience and resistance; such activities lead only to the dissolution of the state. To make this case, Hobbes describes the disobedient as being like “the foolish daughters” in the fable of Pelops. Pelops’s father, Tantalus, wanted to make an offering to the Olympians, and so cut Pelops into pieces and made his flesh into a stew, then served it to the gods in nothing less than a *cauldron of boiling rage*.

The image should not surprise us, because despite the well-known image of Leviathan that appears as the frontispiece of Hobbes’s book of that name—one in which the sovereign is personified in a male figure standing tall, incorporating the subjects and watching over the city—there is, in fact, a much longer and more established image of Leviathan. The image appears in the Book of Job—Hobbes’s favorite Biblical book—as well as the history of demonology. The image is of a creature with flames leaping from his nostrils. It is hard to imagine that Hobbes did not see in this image an expression of sovereign power. The social order becomes nothing less than a cauldron of fear, a raging kettle kept on the boil by the sovereign power, yet always in danger of boiling over into rage and revolt.

The kettle exists to be contained, never to boil over. The kettle thus becomes the *spectacle of a fierce and absolute power*. The police kettle is, in this regard, a microphysics of our political containment—a police tactic to achieve our strategic incapacitation and realise the ultimate point of the Leviathan alluded to by Hobbes through his reference to Job 41:9—that *the hope of man be disappointed*.
